# Supplementary material for: Population-scale study of eRNA transcription reveals bipartite functional enhancer architecture
Source: Nat Commun. 2020 Nov 24;11:5963. doi: 10.1038/s41467-020-19829-z (PMC7687912; doi:10.1038/s41467-020-19829-z)
Supplement: Supplementary file 1 — Supplementary Information [file 41467_2020_19829_MOESM1_ESM.pdf]

Supplemental material for

"Population-scale study of eRNA transcription reveals bipartite functional enhancer architecture"

Authors: Katla Kristjánsdóttir[1,†], Alexis Dziubek[1,†], Hyun Min Kang[2,*], Hojoong Kwak[1,2,*]

Affiliations:

[1]Department of Molecular Biology and Genetics, Cornell University, Ithaca NY, 14853, USA.

[2]Department of Biostatistics, University of Michigan, Ann Arbor, MI 48109, USA.

*Correspondence to Hojoong Kwak (hk572@cornell.edu) or Hyun Min Kang (hmkang@umich.edu)

†These authors contributed equally to this work.

Table of contents:

Supplementary Figures 1-7

Supplementary Tables 1-3

Supplementary Methods

Supplementary References

SUPPLEMENTARY FIGURES

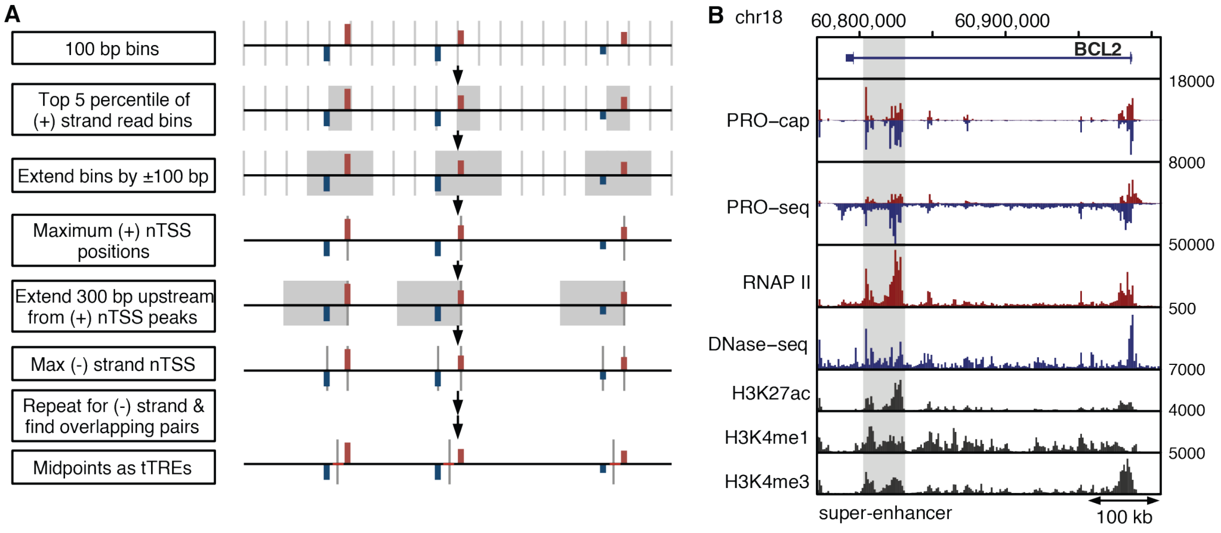

Supplementary Figure 1 | Identification of transcribed transcriptional regulatory elements (tTREs). (A) Schematic of tTRE identification strategy using PRO-cap data. **(B)** Transcription and chromatin marks at the BCL2 locus. PRO-cap, PRO-seq, and DNase-seq data are derived from the YRI LCLs, RNAP II, H3K27, H3K4me2 and H3K4me3 ChIP-seq data are derived from ENCODE’s LCL, GM12878.

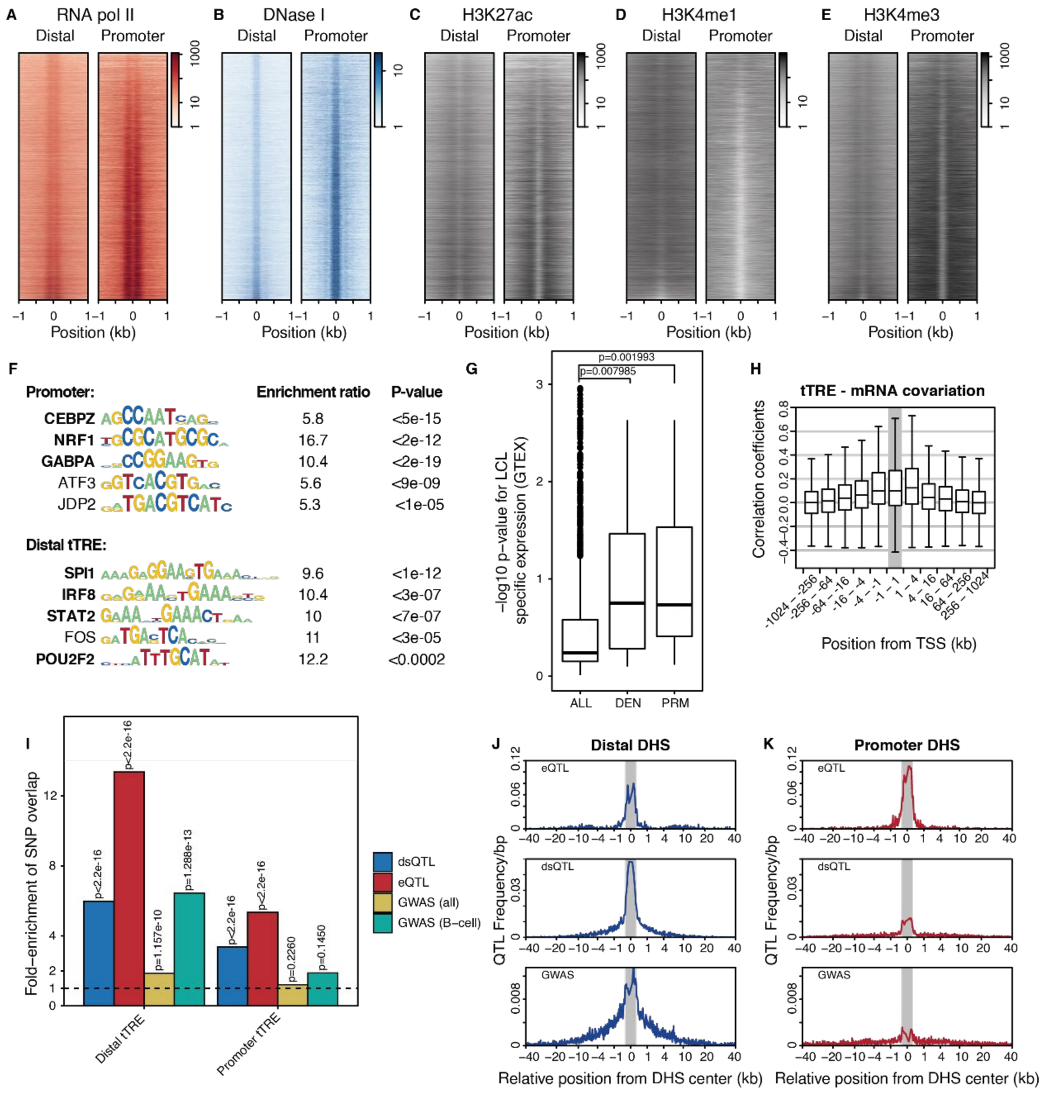

Supplementary Figure 2 | Properties of distal and promoter tTREs. (**A**) Heatmaps of RNA polymerase II density in distal and promoter tTREs. tTREs are ordered according to PRO-cap levels along the y-axis of the heatmaps. Note that highly expressed distal tTREs (bottom) have chromatin landscape comparable to promoter tTREs. (**B**) As in (A) for DNase I density. (**C**) As in (A) for H3K27ac density. (**D**) As in (**A**) for H3K4me1 density. (**E**) As in (A) for H3K4me3 density. (**F**) Motifs enriched at the centers of enhancers and promoters. Top five enriched motifs represented as weblogos. Transcription factors in bold font are lymphoblastoid-specific or have a known blood or immune related function. (**G**) P-value distribution of LCL-specific transcription of TFs for all CISBP motifs (n=3708 motifs) compared to those enriched in promoter (PRM, n=19 motifs) and distal (DEN, n=13 motifs) tTREs. Center line of boxplot indicates the median, box limits are 25th and 75th quantiles, and whiskers are 1.5x interquartile range. Indicated p-values from two-sided Wilcoxon test between groups. (**H**) Covariation between PRO-cap read-counts at tTREs and mRNA expression levels according to both distance and orientation. Pairs were binned based on both the distance between the tTRE and the mRNA TSS, and the orientation of the pairing (TRE upstream: negative numbers, tTRE downstream: positive numbers). The distribution of correlation coefficients in each bin is plotted as a boxplot. Center lines of boxplots indicate medians, box limits are 25th and 75th quantiles, and whiskers are 1.5x interquartile range. (**I**) Overlap enrichment of SNPs at distal and promoter tTREs as compared to background matched SNPs. Indicated p-values from Fisher's exact test. Source data are provided as a Source Data file. (**J**) Enrichment of regulatory variants (expression quantitative trait loci, eQTL; DNase sensitivity QTL, dsQTL; disease associated GWAS SNPs) at distal DNase I hypersensitivity sites (DHS) in comparison to tTREs in Figure 1d. Shaded band: –100 to +100 bp from the DHS window. (**K**) As in (J) for promoter DHS.

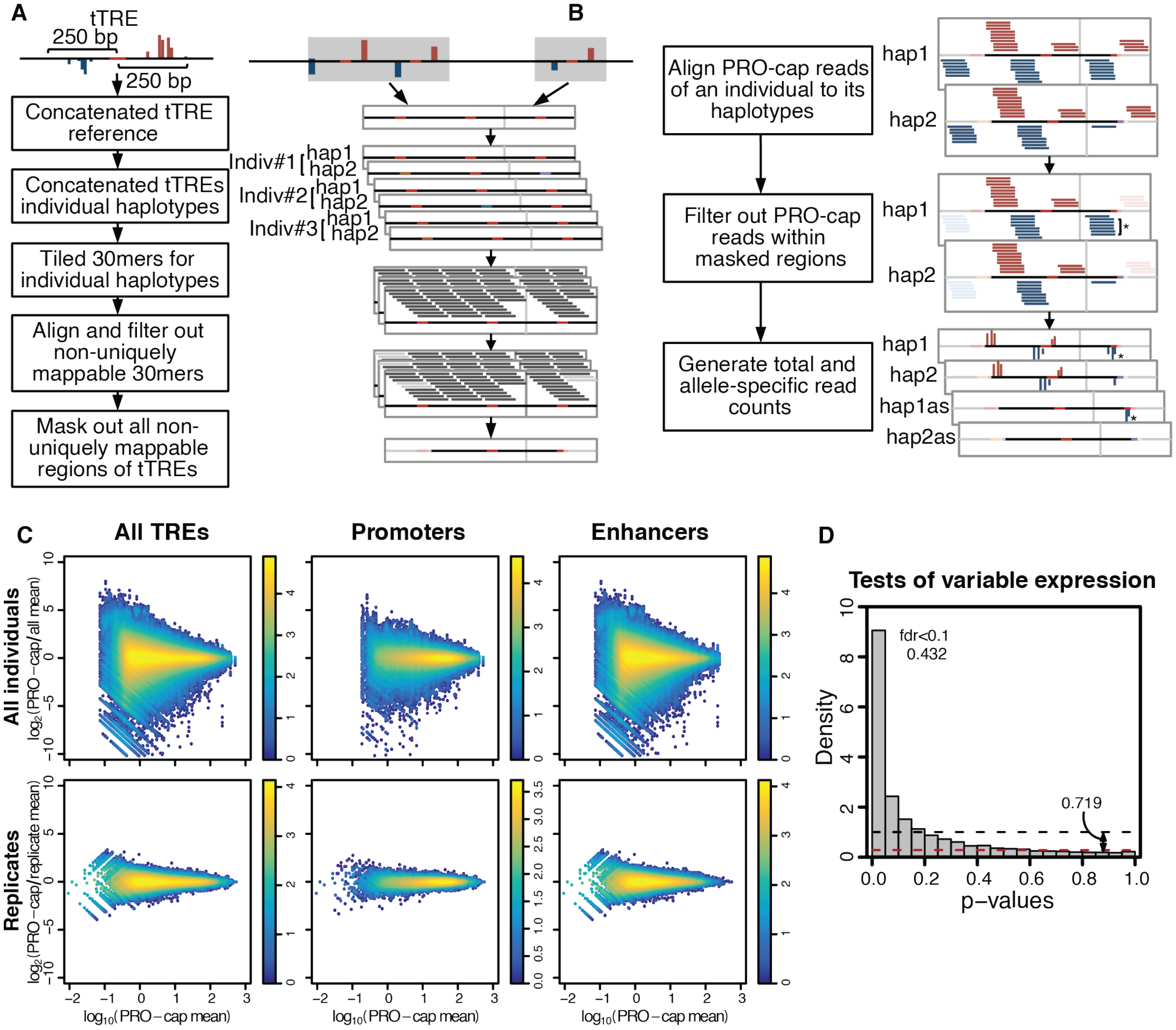

Supplementary Figure 3 | Measurement of the variable tTRE expression across individuals. (A) Schematics of the removal of allele-mappability-biased regions in PRO-cap data alignment. (**B)** Schematics of haplotype specific PRO-cap alignment strategy. (**C)** Scatterplots of PRO-cap variability between individuals in comparison to between replicates. (**D)** Histogram of p-value distribution of variable expression tests in tTREs. The p-values were derived from one-sided Wilcoxon rank-sum tests between the all-individual differences against replicate differences in each tTRE. Estimation of the variably expressed tTRE faction (71.9%) could be derived from the converging p-value density at p→1. About 43% of tTREs can be identified as variably expressed under the false discovery rate = 0.1.

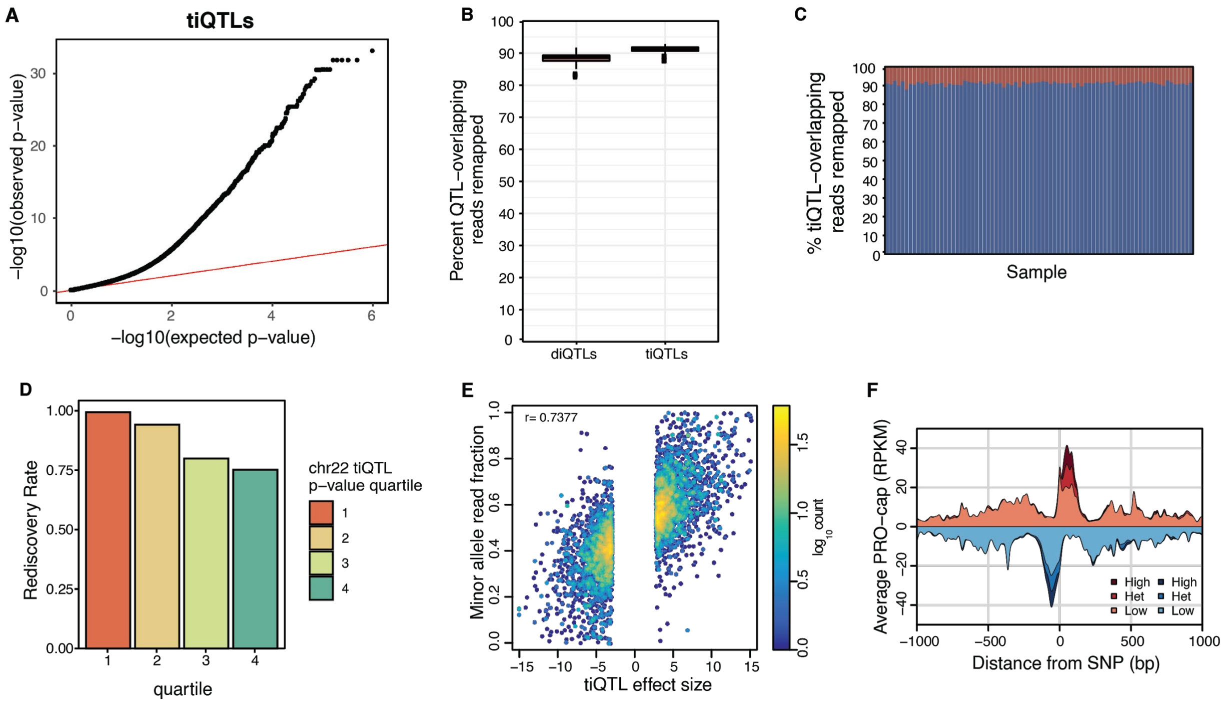

Supplementary Figure 4 | Identified transcription associated QTLs are of high quality (**A**) QQ plot of experimental vs theoretical p-value of all comparisons in tiQTL analysis. Red line indicates the identity line. (**B**) Percent of QTL-overlapping reads that are remapped and not discarded when using WASP. N=79 PRO-cap samples. (**C**) Percent of remapped tiQTL-overlapping reads for each PRO-cap sample. (**D**) Rediscovery rate of tiQTLs after chromosome 22 remapping using WASP. Divided into quartiles based on original tiQTL p-value. (**E**) Allele-specific expression of tiQTL-containing tTREs. The fraction of reads belonging to the minor allele are plotted as a function of the tiQTL effect size. Heatmap indicates number of tiQTLs at coordinate. r is Pearson correlation coefficient. (**F**) Average PRO-cap read-counts surrounding putative causal tiQTLs. Reads for each tiQTL were separated according to genotype into homozygous high-signal allele (High), heterozygous (Het), and homozygous low-signal alleles (Low).

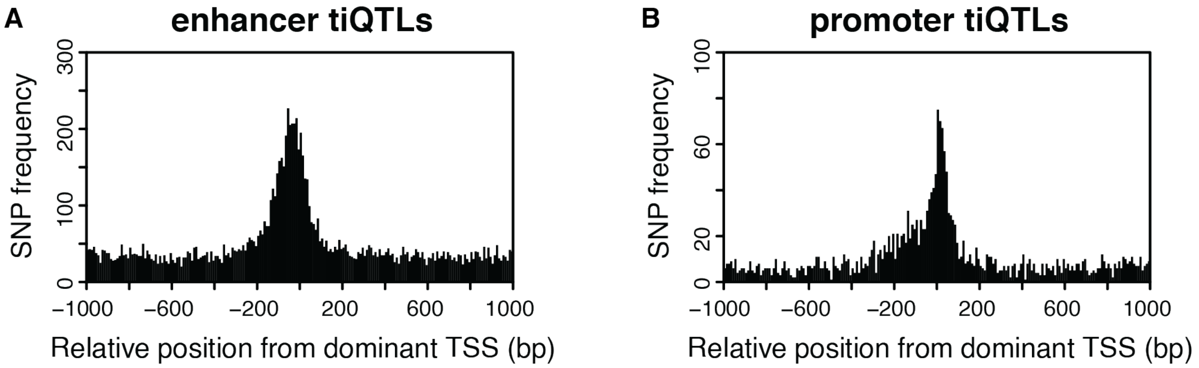

Supplementary Figure 5 | tiQTLs are enriched at enhancer and primary promoter nTSS (**A**) tiQTLs associated with enhancers are enriched upstream of the dominant TSS. A histogram of QTL frequency around enhancer TSS. Enhancers are oriented towards the dominant strand (higher PRO-cap signal). (**B**) tiQTLs affecting promoters are enriched at the dominant TSS. As in (**A**), at promoters.

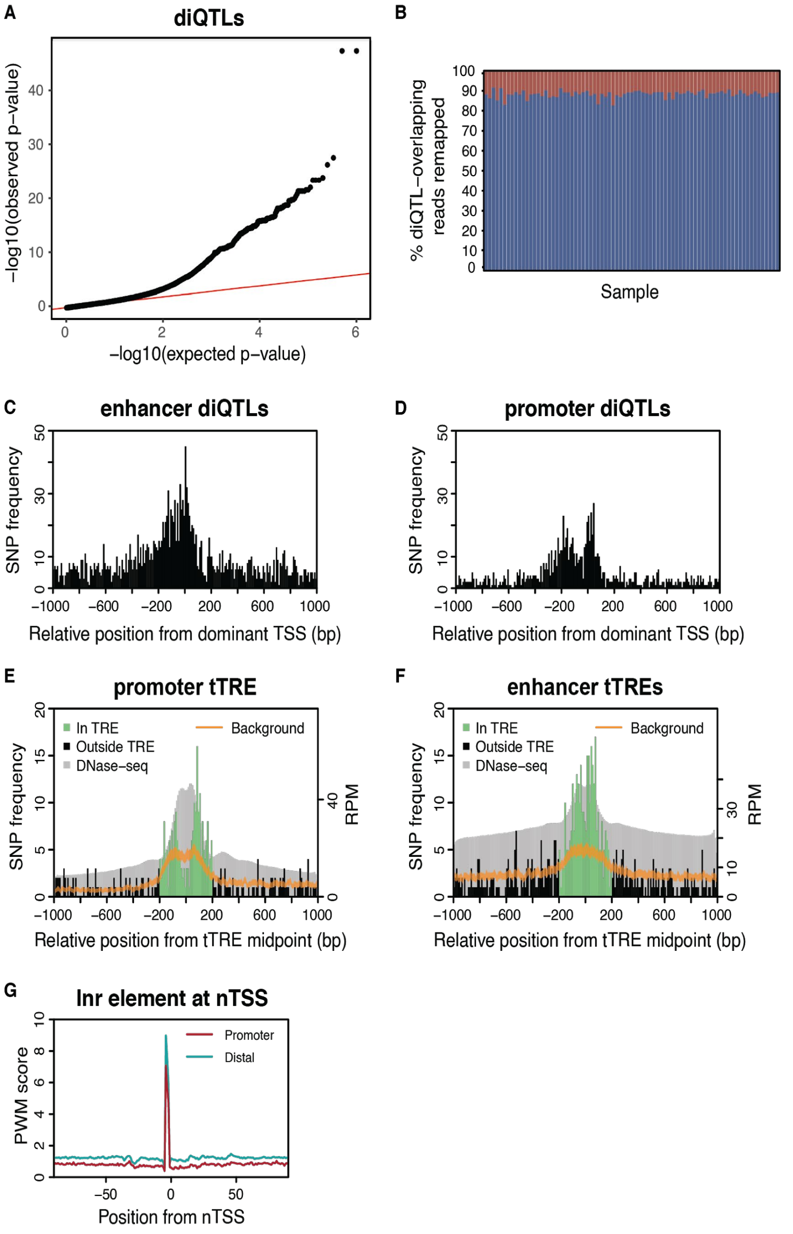

Supplementary Figure 6 | diQTLs are enriched at enhancer and promoter nTSS (**A**) QQ plot of experimental vs theoretical p-value of all comparisons in diQTL analysis. Red line indicates the identity line. (**B**) Percent of remapped diQTL-overlapping reads for each PRO-cap sample. (**C**) diQTLs associated with enhancers are enriched upstream of the dominant TSS. A histogram of QTL frequency around enhancer TSS. Enhancers are oriented towards the dominant strand (higher PRO-cap signal). (**D**) diQTLs affecting promoters are enriched at the dominant TSS. As in (**C**), at promoters. (**E**) A histogram of QTL frequency around enhancer midpoints with the expected background distribution with 99% confidence interval (sampled from all SNPs in same region) shown in orange and aggregate DNase-seq track shown in gray. QTLs are those identified as both diQTLs and tiQTLs. (**F**) As in (**E**), at promoters except oriented so that strand with dominantly transcribed TSS (usually gene) is downstream of the TRE center. (**G**) PWM match scores for Inr are plotted as a function of distance from nTSSs at promoters and distal enhancers.

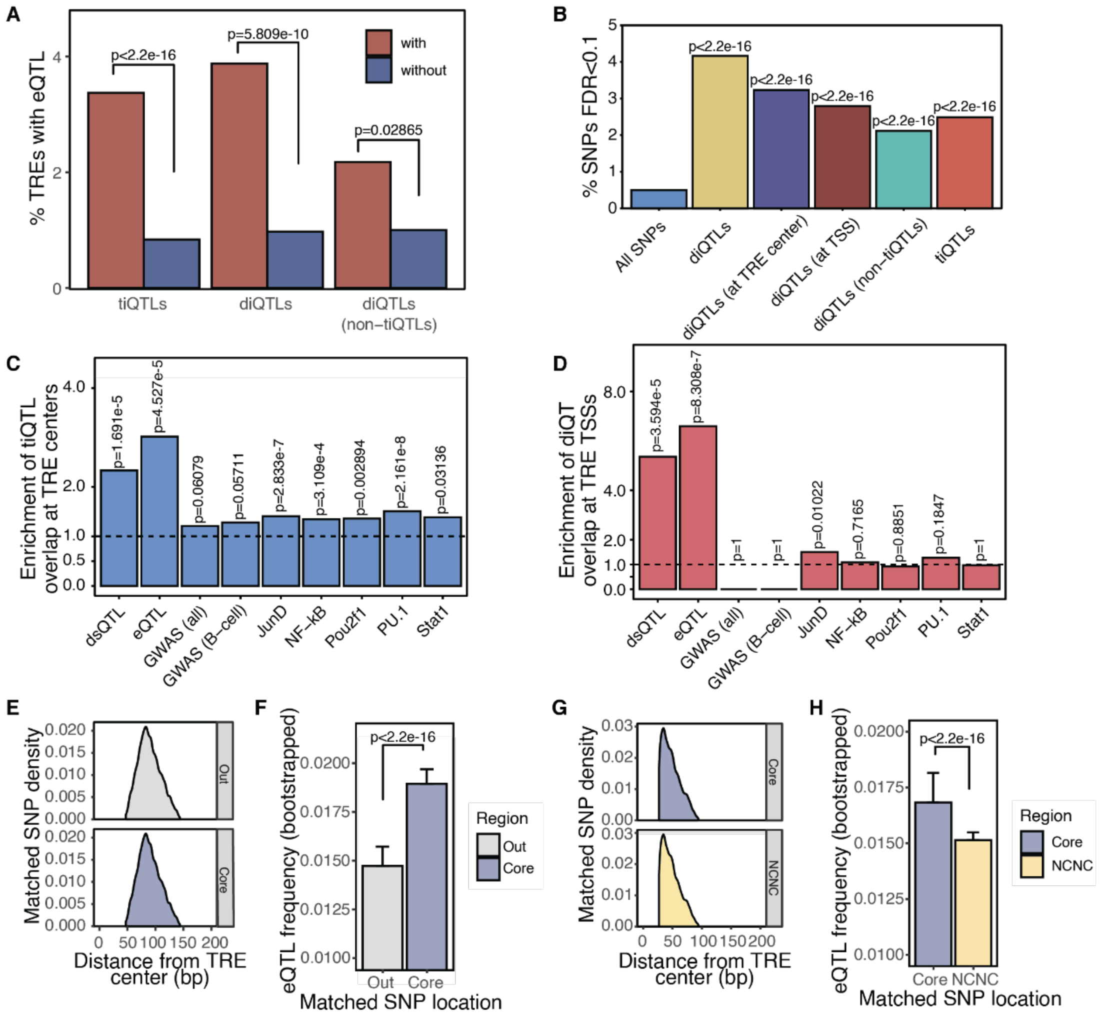

Supplementary Figure 7 | Association of ti- and diQTLs, and intra-TRE regions, with gene expression (**A**) Enhancers that contain tiQTLs and/or diQTLs (with) are enriched in eQTLs, compared to enhancers with no ti- or diQTL (without). The proportion of enhancer tTREs that contain eQTLs were calculated for tiQTL containing, diQTL containing, and exclusively diQTL containing (no tiQTLs) tTREs and compared to those without. Indicated p-values from two-sided Fisher's exact test. Source data are provided as a Source Data File. (**B**) Percent of SNPs with FDR < 0.1 when used as SNP set for eQTL discovery. All p-values calculated in comparison to percentage for all SNPs within 2kb of tTREs. Indicated p-values from two-sidedn Fisher's exact test. Source data are provided as a Source Data file. (**C**) Enrichment of tiQTLs +/- 20 bp from TRE centers compared to overlap of background SNPs. Overlap of QTL and background are compared for each bar with two-sided Fisher's exact test. Source data are provided as a Source Data file. (**D**) As in (**C**) for diQTLs at TRE TSSs +/- 25 bp. Source data are provided as a Source Data file. (**E**) Frequency of eQTLs among distance-from-center-matched SNPs from out and core regions of enhancer tTREs. Left: SNP density plotted as function of distance from TRE center. (**F**) Bootstrapped frequency of eQTLs in (**E**). Data are presented as mean eQTL frequency +/- standard deviation. Indicated p-value from bootstrapped Welch's two-sided t-test, n = 500 bootstrapped eQTL frequencies. Source data are provided as a Source Data file. (**G**) Frequency of eQTLs among distance-from-center-matched SNPs from core and non-core non-center regions of enhancer tTREs. Left: SNP density plotted as function of distance from TRE center. (**H**) Bootstrapped frequency of eQTLs in (**G**). Data are presented as mean eQTL frequency +/- standard deviation. Indicated p-value from bootstrapped Welch's two-sided t-test, n = 500 bootstrapped eQTL frequencies. Source data are provided as a Source Data file.

Supplementary Tables

Supplementary Table 1: Promoter tTRE motif enrichment results and FDR

No.	Motif ID	TF Name	N. Pos.	Expected.	p-value	*Enrichment	Motif Logo
1	M5680_1.02	NR2F6	9	431.5	<9e-18	0	
2	M6174_1.02	CEBPZ	1556	269.7	<5e-15	5.8	
3	M2960_1.02	NR2F2	5	296.6	<4e-13	0	
4	M5688_1.02	NRF1	903	53.9	<2e-12	16.7	
5	M4505_1.02	GABPA	841	80.9	<2e-10	10.4	
6	M4569_1.02	HSF1	10	269.7	<4e-10	0	
7	M6409_1.02	PAX5	59	458.5	<1e-09	0.1	
8	M4500_1.02	ATF3	901	161.8	<9e-09	5.6	
9	M3524_1.02	TOPORS	7	188.8	<2e-07	0	
10	M6522_1.02	THRA	9	188.8	<6e-07	0	
11	M5974_1.02	ZNF524	18	215.7	<2e-06	0.1	
12	M5588_1.02	JDP2	577	107.9	<1e-05	5.3	
13	M6537_1.02	YBX1	329	0	<1e-05	12.2	
14	M2942_1.02	KLF12	48	269.7	<3e-05	0.2	
15	M6483_1.02	SP4	291	0	<4e-05	10.8	
16	M6463_1.02	SMAD1	3	107.9	<5e-05	0	
17	M6273_1.02	HEY2	1	80.9	<0.0002	0	
18	M6181_1.02	CREM	461	107.9	<0.0003	4.3	
19	M4594_1.02	CTCF	226	0	<0.0004	8.4	
20	M5875_1.02	TBX1	24	161.8	<0.0006	0.1	
21	M0305_1.02	CREB3L2	199	0	<0.001	7.4	
22	M3720_1.02	PAX5	7	80.9	<0.005	0.1	
23	M6551_1.02	ZNF143	274	53.9	<0.005	5.1	
24	M6553_1.02	ZNF219	16	107.9	<0.005	0.1	
25	M4484_1.02	ZNF143	524	215.7	<0.006	2.4	
26	M5787_1.02	RORA	8	80.9	<0.006	0.1	
27	M6488_1.02	SREBF2	20	107.9	<0.01	0.2	
28	M6453_1.02	RFX3	141	0	0.01	5.2	
29	M6147_1.02	ARID3A	406	701.2	0.01	0.6	
30	M4692_1.02	SIX5	382	134.8	0.01	2.8	
31	M6251_1.02	FUBP1	168	350.6	0.01	0.5	
32	M4012_1.02	CREB1	132	0	0.02	4.9	
33	M5436_1.02	FOXB1	70	188.8	0.02	0.4	
34	M4489_1.02	SPI1	309	107.9	0.02	2.9	
35	M5864_1.02	SPIB	123	0	0.02	4.6	
36	M2943_1.02	TFAP4	16	80.9	0.03	0.2	
37	M6509_1.02	TEAD4	62	161.8	0.03	0.4	
38	M0428_1.02	ZNF691	0	27	0.04	0	
39	M0632_1.02	DMRTA2	0	27	0.04	0	
40	M5946_1.02	VDR	0	27	0.04	0	
41	M6222_1.02	ETV4	438	215.7	0.04	2	
42	M6286_1.02	HSF1	18	80.9	0.04	0.2	
43	M4635_1.02	STAT2	247	80.9	0.04	3.1	
44	M4487_1.02	PAX5	8	53.9	0.05	0.1	

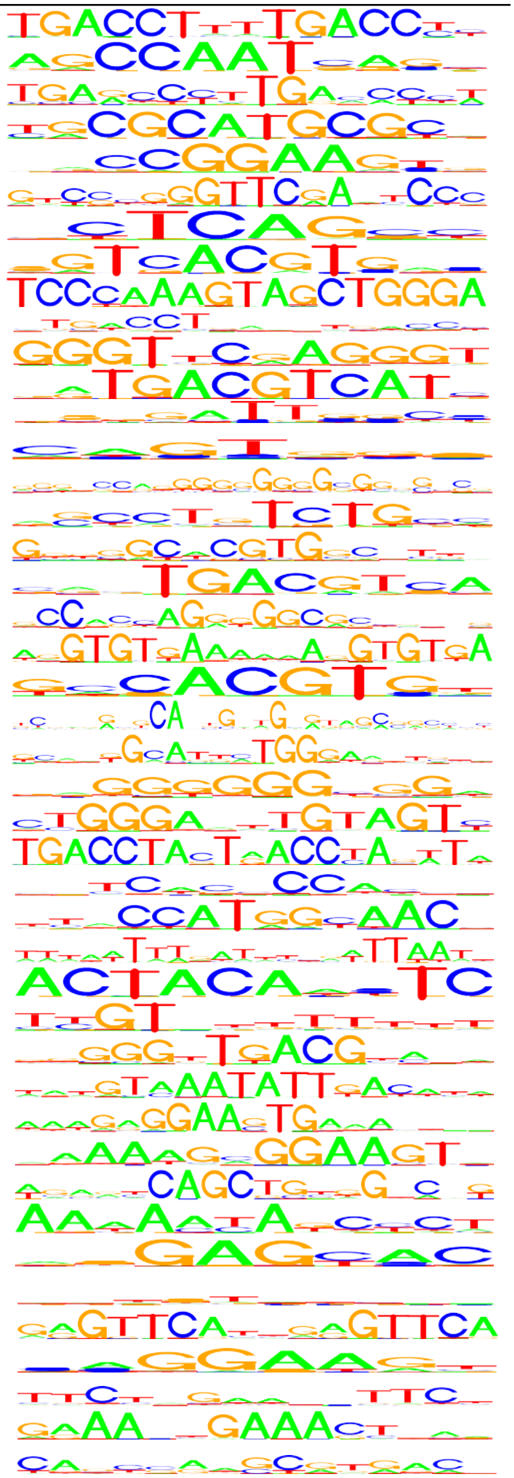

*Enrichment: Enrichment ratio for positive reads against negative reads.

Table showing significant motifs with enrichment and depletion of transcription factor binding sites (n=400 representative transcription factor motifs) in promoter tTREs over a background set. P-values are all Bonferroni corrected two-sided Fisher's exact test values.

Supplementary Table 2: Enhancer tTRE motif enrichment results and FDR

No.	Motif ID	TF Name	N. Pos.	Expected.	p-value	*Enrichment	Motif Logo
1	M4489_1.02	SPI1	2608	272.9	<1e-12	9.6	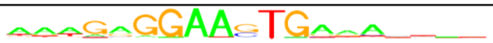
2	M6313_1.02	IRF8	1419	136.5	<3e-07	10.4	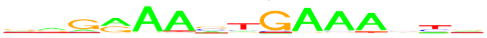
3	M4635_1.02	STAT2	1358	136.5	<7e-07	10	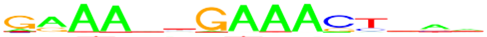
4	M4530_1.02	FOS	748	0	<3e-05	11	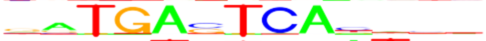
5	M4486_1.02	POU2F2	832	68.2	<0.0002	12.2	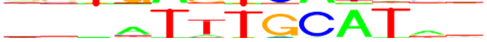
6	M6522_1.02	THRA	101	545.8	<0.0002	0.2	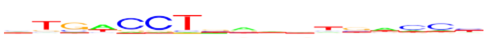
7	M6552_1.02	ZNF148	1836	682.3	<0.0003	2.7	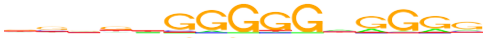
8	M6539_1.02	ZBTB7B	1849	682.3	<0.0003	2.7	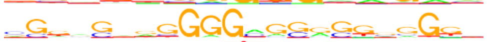
9	M3617_1.02	NFE2	590	0	<0.0003	8.6	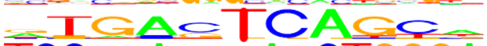
10	M3524_1.02	TOPORS	58	341.1	<0.002	0.2	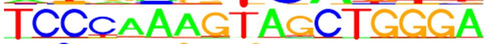
11	M4594_1.02	CTCF	867	204.7	<0.002	4.2	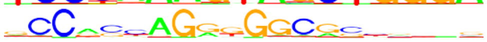
12	M6483_1.02	SP4	4519	3002.1	<0.003	1.5	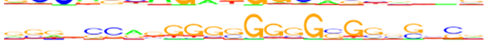
13	M6147_1.02	ARID3A	310	818.8	<0.003	0.4	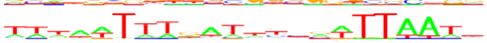
14	M5680_1.02	NR2F6	67	341.1	<0.004	0.2	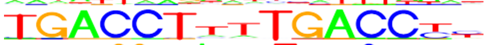
15	M3784_1.02	PPARG	5	136.5	<0.004	0	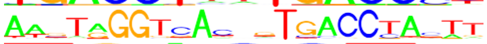
16	M6443_1.02	RARA	30	204.7	0.01	0.1	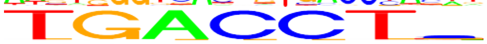
17	M5879_1.02	TBX1	10	136.5	0.01	0.1	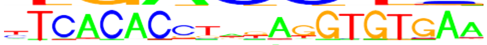
18	M4545_1.02	PRDM1	505	68.2	0.01	7.4	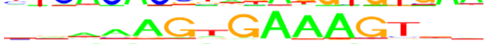
19	M6242_1.02	FOXJ3	36	204.7	0.02	0.2	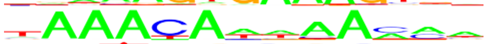
20	M1359_1.02	MYPOP	36	204.7	0.02	0.2	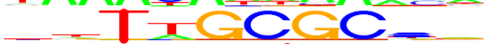
21	M6174_1.02	CEBPZ	691	204.7	0.02	3.4	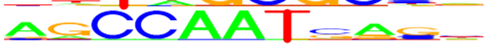
22	M5333_1.02	CUX1	16	136.5	0.03	0.1	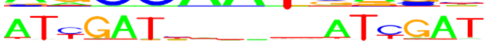
23	M5346_1.02	DPRX	18	136.5	0.03	0.1	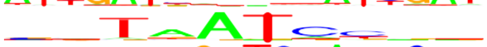
24	M4569_1.02	HSF1	48	204.7	0.04	0.2	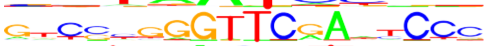
25	M4500_1.02	ATF3	371	68.2	0.05	5.4	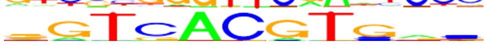

*Enrichment: Enrichment ratio for positive reads against negative reads

This table shows enrichment ratios and p-values as in Supplementary Table 1 for distal enhancer tTREs.

Supplementary Table 3: B-cell related GWAS traits | This table contains the GWAS traits and ontology identifiers used to filter for B-cell specific GWAS SNPs.

GWAS Trait	Ontology Identifier
diffuse large b-cell lymphoma	EFO_0000403
B-cell acute lymphoblastic leukemia	EFO_0000094
marginal zone B-cell lymphoma	EFO_1000630
neoplasm of mature b-cells	EFO_0000096
common variable immunodeficiency	Orphanet_1572
multiple myeloma	EFO_0001378
selective IgA deficiency disease	EFO_1001929
Waldenstrom macroglobulinemia	EFO_0009441
central nervous system non-hodgkin lymphoma	MONDO_0044887
follicular lymphoma	MONDO_0018906
chronic lymphocytic leukemia	EFO_0000095
HOMA-B	EFO_0004469
CXCL13 measurement	EFO_0009421
response to immunochemotherapy	EFO_0007754
acute lymphoblastic leukemia	EFO_0000220
event free survival time	EFO_0000482
lymphoma	EFO_0000574
systemic lupus erythematosus	EFO_0002690
rheumatoid arthritis	EFO_0000685
multiple sclerosis	EFO_0003885
hodgkins lymphoma	EFO_0000183

Supplementary methods:

Alignment of PRO-cap/PRO-seq reads to the reference genome.

For each dataset, we pre-processed the raw sequence reads (fastq) by trimming out 3′ RNA adaptor sequences using cutadapt, and tagging the reads with first 8mer of the sequences for unique molecular indices (UMIs). We aligned the reads to a human ribosomal RNA reference (NR_145819) and filtered out the reads that mapped to the ribosomal sequences using bowtie, allowing up to 2 mismatches. Then we aligned the remaining reads to the hg19 reference genome using bowtie, and used uniquely mapped reads allowing up to 2 mismatches. Duplicate reads, having the same mapped position and the same UMI, were reduced to a single read using custom scripts, and stored in bam format. We mapped the 5′ (PRO-cap) or 3′ (PRO-cap) ends of the reads in each individual and stored the read counts in bedgraph format using the bedtools suite (bedtools coverage).

Identification of nTSSs and tTREs.

To find nascent transcription start sites (nTSSs), we made 100 bp bins of the PRO-cap read counts at the 5′ end of the reads on the plus strands of the hg19 genome, and selected the top 5 percentile bins (1.5 million bins). To find bidirectional PRO-cap peaks in these bins, we extended the top 1.5 million 100 bp bins by 100 bps upstream and downstream, and picked the 1 bp position with the highest read count greater than 5 reads within the resulting 300 bp region. We then selected antisense strand peaks that have the highest read counts (greater than 5 reads) within 300 bp upstream relative to the sense strand peak. We repeated this procedure starting with the minus strand and combined both to generate 491,289 bidirectional PRO-cap peak candidates.

Since transcription initiation can arise from a broader region up to several tens of base pairs, we made sums of PRO-cap read counts from the midpoint to +200 bp on the plus strand, and -200 bp to the midpoint on the minus strand, for each PRO-cap peak candidates. Then we removed PRO-cap peaks whose midpoints are within 150 bp of another peak's midpoint, keeping only the peak with the highest read counts (219,312 PRO-cap peaks). The read count sums for each PRO-cap peak were normalized by per million mapped reads (RPM).

To select PRO-cap peaks that are expressed above the background distribution of PRO-cap read counts, we repeated our analysis on randomly selected 1,000,000 genomic positions that are 1 kb away from annotated TSSs. We could then calculate the p-values for the 219,312 PRO-cap peaks under the random background assumption. We chose a cut-off of 0.5 RPM, which corresponds to a p-value of 0.0067 (fdr=0.017) under this background estimation (n=87,826; **Supplementary Figure 1A**). In these PRO-cap peaks, we defined each of the strand specific PRO-cap peaks as the nascent transcription start sites (nTSSs) and the 400 bp bidirectional peak regions as the transcribed transcriptional regulatory elements (tTREs). The coordinates and other PRO-cap measurements at the tTRE are collectively provided in **Supplementary Table 2**.

Variant sensitive alignment of PRO-cap reads.

We generated concatenated tTRE sequences covering the tTRE regions using the phased haplotype data of the 67 individuals we tested from the HapMap III database. We extracted hg19 reference sequences from -250 bp to + 250bp (200 bases + 50 base read length) of the 87,826 nTSS midpoints, merged overlapping regions, and concatenated these regions with 100 bp paddings (N's) to generate the tTRE reference. Then we used HapMap variant calls to modify sequences to generate two tTRE haplotypes for each individual (67 individuals, 134 haplotypes, ~40.6 Mbases for each haplotype). We also incorporated short indels to the haplotype sequences.

A total of 241,176 variant positions were used in the tTRE regions. We transformed the coordinates of the tTRE regions and SNP positions for every individual concatenated tTRE haplotype sequences coordinates in case the indels changed the coordinates.

To mask out and exclude non-unique regions for further analyses, we generated tiled 30mer reads from the concatenated individual tTRE haplotype DNA sequences and assessed their unique mappability. We mapped the reads back to the hg19 reference genome and to the two tTRE haplotype sequences from the same individual. We removed three types of non-uniquely mappable tiled 30mers: 1) 30mers from the tTRE reference that mapped to more than 1 position in the hg19 genome allowing up to 2 mismatches - removes PRO-cap reads that are originally from non-tTRE regions but are mismapped to the tTRE regions, 2) 30mers where the alternative variant of the individual tTRE haplotype sequences mapped to more than 1 position on the hg19 genome allowing up to 2 mismatches - removes PRO-cap reads originally from non-tTRE regions being mismapped to the tTRE regions with variant alleles, 3) 30mers from the heterogeneous variant sites of the individual tTRE haplotype sequences that mapped to any other regions of the two tTRE haplotype sequences in that individual - removes PRO-cap reads from tTRE regions that can be mismapped to other tTRE regions. We assembled these 3 types of regions across all individuals and generated a common non-mappable position list. Then we transformed these non-mappable positions to the individual tTRE haplotype coordinates, and masked out these regions.

The PRO-cap reads were aligned to the two tTRE haplotype sequences of the same individuals separately allowing only perfect matches. Reads with the same UMIs mapped to the same coordinates are collapsed to a single read. We removed reads whose 5′ and 3′ ends are both within masked non-mappable regions. Allele specific PRO-cap reads will be mapped only once

to the heterogeneous variant sites of one of the two tTRE haplotype sequences, while non-allele specific PRO-cap reads will be mapped to both haplotypes. We extracted reads that are covering the heterogeneous variant sites as the allele specific reads. We then generated total read counts and allele specific read counts for each haplotype for every tTRE regions (0 - +250 relative to the tTRE midpoints for the plus strand, -250 – 0 relative to the tTRE midpoints for the minus strand). Total read counts were half the sum of read counts on haplotype 1, read counts on haplotype 2, allele specific read counts on haplotype 1, and allele specific read counts on haplotype 2.

Validation of tiQTLs using allele specific expression. For the allele specific analysis, we used 28,118 tiQTLs (fdr<0.1) whose SNP sites are within -250 - +250 bp from the tTRE midpoints. Of these sites, we used the 5,317 sites that have more than 90 allele specific PRO-cap reads, and plotted the alternative allele read fractions (y-axis) as a function of the tiQTL effect sizes (x-axis) (**Supplementary Fig. 4A**).

Validation of tiQTL and diQTL using WASP. We used WASP[1] mappability filtering pipeline as described in the tool suite (https://github.com/bmvdgeijn/WASP). Since WASP filtering was a computationally intense procedure, we applied WASP filtering test only on the reads that intersect with the tiQTL or diQTL SNPS, which were 34.2 million and 9.98 million reads respectively from the 67 LCL PRO-cap data rather than all the 1.4 billion reads. After mapping back to the genome post-filtering by WASP, we calculated the re-mapping fraction of the tiQTL and diQTL mapped reads per each individual sample. We also applied WASP filtering on all the reads that mapped to chromosome 22 in our pipeline, and re-tested the tiQTL associations at the same adjusted p-values thresholds to evaluate the re-discovery rate.

Average profiles of tiQTL effects. We selected the tiQTLs most likely to be causal variants: These tiQTLs 1) are significant at FDR<0.1 in 2 kb analysis, 2) the lead SNP is located in the target tTRE midpoint ±40 bp region, 3) and the lead SNP is 10 times more significantly associated (p-value 10 times smaller) than the 2nd SNP (n=1,226). We extracted RPKM normalized PRO-cap read profiles from ±1 kb regions around the SNPs for each individual, assigned them according to the individual's genotype class (high-activity/heterogeneous/low-activity), and averaged the genotype class both the plus and the minus strands. The averaged profiles were fit to smoothing splines (R smooth.spline function with spar=0.3, **Supplementary Fig. 4B**).

Distance-matched bootstrapping of eQTLs. SNPs were sampled from the core region and either the out or NCNC region, maintaining an equivalent distribution of distances from the tTRE midpoint between the samples. The resulting sample sizes are N = 5088 SNPs for core vs. out and N = 4128 for core vs. NCNC and samplings are repeated 500 times. Each time the proportion of SNPs that pass the fdr < 0.05 threshold for gene expression association is computed (eQTL proportion). The mean and standard deviation of the 500 eQTL proportions was calculated, and a bootstrap-estimated P-value was computed by the fraction of the 500 eQTL proportions for which the core had a higher proportion than the mean for the comparison group (out or NCNC).

Supplementary References

1. van de Geijn B, McVicker G, Gilad Y, Pritchard JK. WASP: allele-specific for robust molecular quantitative trait locus discovery. *Nat Methods*. **12**, 1061–3 (2015).
