# Supplementary material for: Population-scale study of eRNA transcription reveals bipartite functional enhancer architecture
Source: Nat Commun. 2020 Nov 24;11:5963. doi: 10.1038/s41467-020-19829-z (PMC7687912; doi:10.1038/s41467-020-19829-z)
Supplement: Supplementary file 3 — Reporting Summary [file 41467_2020_19829_MOESM3_ESM.pdf]

nature research

Corresponding author(s): Hojoong Kwak, Hyun Min Kang

Last updated by author(s): Oct 23, 2020

Reporting Summary

Nature Research wishes to improve the reproducibility of the work that we publish. This form provides structure for consistency and transparency in reporting. For further information on Nature Research policies, see our Editorial Policies and the Editorial Policy Checklist.

Statistics

For all statistical analyses, confirm that the following items are present in the figure legend, table legend, main text, or Methods section.

n/a	Confirmed	
☐	☒	The exact sample size (n) for each experimental group/condition, given as a discrete number and unit of measurement
☐	☒	A statement on whether measurements were taken from distinct samples or whether the same sample was measured repeatedly
☐	☒	The statistical test(s) used AND whether they are one- or two-sided<br>*Only common tests should be described solely by name; describe more complex techniques in the Methods section.*
☐	☒	A description of all covariates tested
☐	☒	A description of any assumptions or corrections, such as tests of normality and adjustment for multiple comparisons
☐	☒	A full description of the statistical parameters including central tendency (e.g. means) or other basic estimates (e.g. regression coefficient) AND variation (e.g. standard deviation) or associated estimates of uncertainty (e.g. confidence intervals)
☐	☒	For null hypothesis testing, the test statistic (e.g. F, t, r) with confidence intervals, effect sizes, degrees of freedom and P value noted<br>*Give P values as exact values whenever suitable.*
☒	☐	For Bayesian analysis, information on the choice of priors and Markov chain Monte Carlo settings
☒	☐	For hierarchical and complex designs, identification of the appropriate level for tests and full reporting of outcomes
☐	☒	Estimates of effect sizes (e.g. Cohen's d, Pearson's r), indicating how they were calculated

Our web collection on statistics for biologists contains articles on many of the points above.

Software and code

Policy information about availability of computer code

Data collection	No software used for the data collection
Data analysis	We used bash v3.2, fastq, cutadapt, bowtie, R v3.6.l, DESeq2 v1.28 , ggplot2 v3.2.l, pixmap v0.4.11, tidyverse vl.3.0, dplyr v0.8 .3, ggpubr v0.2.4, GenomicRanges vl.36.1, tidyr vl.0.0, rlang v0.4.1, bedtools v2.26.0, RTFBSdb v0.4.0, M atrixEQTL v2.2, HTSlib vl.3.1, GAT vl.3.5, SNPsnap, and motifbreakR vl.14.0 for analysis, as well as custom scripts which identifyTREs and produce a variant sensitive alignment of our reads to each genome.

For manuscripts utilizing custom algorithms or software that are central to the research but not yet described in published literature, software must be made available to editors and reviewers. We strongly encourage code deposition in a community repository (e.g. GitHub). See the Nature Research guidelines for submitting code & software for further information.

Data

Policy information about availability of data

All manuscripts must include a data availability statement. This statement should provide the following information, where applicable:

- Accession codes, unique identifiers, or web links for publicly available datasets
- A list of figures that have associated raw data
- A description of any restrictions on data availability

The sequencing libraries from this study have been submitted to the NCBI Gene Expression Omnibus (GEO) with accession number GSE110638 (reviewer token to view while private: alczwuoqfxsjbav). All the data bases used in the study are listed in the Data availability section with accessible links including DNA-seq, RNA-seq studies in relevant cell types, and reference genomic annotaion data - hg19 reference genome and gene annotation, GWAS catalog, and ENCODE ChIP-seq data.

Field-specific reporting

Please select the one below that is the best fit for your research. If you are not sure, read the appropriate sections before making your selection.

[x] Life sciences [ ] Behavioural & social sciences [ ] Ecological, evolutionary & environmental sciences

For a reference copy of the document with all sections, see nature.com/documents/nr-reporting-summary-flat.pdf

Life sciences study design

All studies must disclose on these points even when the disclosure is negative.

Sample size	Lymphoblastoid cell lines (LCLs) from 70 de-identified individuals provided through HapMap consortium. Sample size were determined to be consistent with an existing study by Degner et al. (Nature 482, 390–394 ,2012)
Data exclusions	1 individual was not included in all of the analysis (due to experimental failure), resulting the total size of 69. Data from 2 individuals was not included in the ti/diQTL analysis because they didn't have phased genotype data.
Replication	A subset of the PRO-cap samples and all of the PRO-seq samples had a second replicate performed which have lower variability between replicates compared to between individuals, as expected.
Randomization	When processing batches of samples, cell lines were randomly picked to be processed together. All cells were treated the same, so there was no need to divide them into treatment or control groups.
Blinding	Blinding was not relevant to the study as all samples for PRO-cap and PRO-seq were treated the same way and run through the same protocol. Group allocation were randomized, but not completely blinded whereas only the sample identifier number of the LCLs were exposed to the researcher.

Reporting for specific materials, systems and methods

We require information from authors about some types of materials, experimental systems and methods used in many studies. Here, indicate whether each material, system or method listed is relevant to your study. If you are not sure if a list item applies to your research, read the appropriate section before selecting a response.

Materials & experimental systems

n/a	Involved in the study
[x]	[ ] Antibodies
[ ]	[x] Eukaryotic cell lines
[x]	[ ] Palaeontology and archaeology
[x]	[ ] Animals and other organisms
[x]	[ ] Human research participants
[x]	[ ] Clinical data
[x]	[ ] Dual use research of concern

Methods

n/a	Involved in the study
[x]	[ ] ChIP-seq
[x]	[ ] Flow cytometry
[x]	[ ] MRI-based neuroimaging

Eukaryotic cell lines

Policy information about cell lines

Cell line source(s)	Coriell Biorepository, Lymphoblastoid cell lines GM18486 etc (details listed in the supplementary information)
Authentication	None
Mycoplasma contamination	Not tested
Commonly misidentified lines (See ICLAC register)	No commonly misidentified cell lines were used in the study.
