# Supplementary material for: Population-scale study of eRNA transcription reveals bipartite functional enhancer architecture
Source: Nat Commun. 2020 Nov 24;11:5963. doi: 10.1038/s41467-020-19829-z (PMC7687912; doi:10.1038/s41467-020-19829-z)
Supplement: Supplementary file 4 — Description of Additional Supplementary Files [file 41467_2020_19829_MOESM4_ESM.pdf]

Description of Additional Supplementary Files

File Name: Supplementary Data 1
Description: Description of Lymphoblastoid Cell Lines (LCLs) used in this study. Columns indicate de-identified individual id (cell line names), PRO-cap experimental batch number, PRO-cap replicate batch number if replicate experiment is performed, PRO-seq batch number if PRO-seq experiment is performed, PRO-seq replicate batch if any, availability of HapMap III phased genotype, and PROcap/PRO-seq raw and processed data availabilities. Asterisks indicate abnormalities in the experiment such as library failure or cross contamination using a contamination detection software package (http://genome.sph.umich.edu/wiki/ContaminationDetection).

File Name: Supplementary Data 2
Description: List of transcribed Transcription Regulatory Elements (tTREs) identified from LCLs. Columns indicate genomic coordinates, properties of the tTRE, allele mappability of the region within the individual genotypes we used, presence of PRO-cap expression variations, and normalized PRO-cap levels in the 67 LCLs (+ 9 replicated PRO-cap data). PRO-cap levels are 0 if the PRO-cap levels of the tTRE are not robustly measurable across the individuals due to the allele mappability bias.
